# Estimation Method of Interpass Time for the Control of Temperature during a Directed Energy Deposition Process of a Ti–6Al–4V Planar Layer

**DOI:** 10.3390/ma13214935

**Published:** 2020-11-03

**Authors:** Bih-Lii Chua, Dong-Gyu Ahn

**Affiliations:** 1 Faculty of Engineering, Universiti Malaysia Sabah, Kota Kinabalu 88400, Malaysia; bihlii@ums.edu.my; 2 Department of Mechanical Engineering, Chosun University, 309 Pilmun-daero, Dong-gu, Gwangju 61452, Korea

**Keywords:** interpass time, thermal stress, additive manufacturing, directed energy deposition, Ti–6Al–4V, planar layer, thermo-mechanical analysis

## Abstract

Directed energy deposition (DED) provides a promising additive manufacturing method to fabricate and repair large metallic parts. However, it may suffer from excessive heat accumulation due to a high build rate, particularly during a wire feeding-type DED process. The implementation of interpass time in between two depositions of beads plays an important process role to passively control the interpass temperature. In this study, a method to estimate the proper interpass time using regression analysis from heat transfer finite element analysis is proposed for maintaining the interpass temperature during a wire feeding-type DED deposition of a planar layer. The overlapping beads of a planar layer are estimated using a polygonal-shaped bead profile in the finite element model. From the estimated proper interpass time, a selected proper interpass time scheme (PITS) is suggested for practical implementation. The selected PITS is applied in a thermo-mechanical finite element model to evaluate the temperature distribution and its effects on the depth of the melt pool, the depth of the heat-affected zone (HAZ), displacement, and residual stresses. By comparing the predicted results with those using a constant interpass time scheme (CITS), the selected PITS shows better control in reducing the depths of the melt pool and HAZ without severely inducing large displacement and residual stresses.

## 1. Introduction

Many industrial products use metals to form components or structures to meet their functional and aesthetic requirements. In the era of the Industrial Revolution 4.0, metal additive manufacturing plays an important role to complement the conventional manufacturing techniques. Directed energy deposition (DED) is a metal additive manufacturing technology that uses a high-intensity heat source to melt and fuse metallic feedstock to substrate as they are being fed. It has shown promising applications for the production of large metallic components and the repair of the damaged region of an existing part [1,2,3]. In the case of remanufacturing and repairing a component, molding, or forming tool using the DED process [4,5,6,7,8], the ability to selectively deposit planar layers on the designated location of an existing substrate is crucial. This required feature distinguishes the DED process from a multi-pass cladding process. With proper process parameters such as laser power, traverse speed, and substrate temperature, a high quality of hard and wear resistance material can be additively manufactured [9]. A planar layer can be augmented to the existing substrate by depositing tracks of metals side by side [3,4]. A three-dimensional object is fabricated by stacking multiple planar layers in a vertical build direction. It is expected that high value large-scale industries such as aerospace and automotive will benefit significantly from the development of DED [3].

Nevertheless, industrial production using DED is considered premature. The identification of process parameters is crucial to fabricate a product with required quality [10]. This challenge is rooted in the application of enormous heat input for melting the metallic feedstock, especially in the form of wire. In addition, a large temperature gradient during the deposition process may result in significant distortion and detrimental residual stresses induced in the components [1]. Adversely, it leads to undesirable loss in the dimensional accuracy, mechanical strength, and toughness of the final product. In order to alleviate these problems, heat transfer-based process control is required.

Investigation of the influencing factors using numerical analyses has recently become prominent to significantly reduce the number of experimental trial-and-error run in the search for the optimum process parameters of a DED process [3,5,11,12,13]. These process parameters are determined based on simulation results of one or more combinations of temperature distributions [14,15,16], thermo-mechanical characteristics [17,18,19,20,21], and microstructure evolution [1,22]. Piscopo et al. [10] developed a finite element model to predict the track geometry and temperature distributions according to the laser power, powder feed rate, and scanning speed during a powder feeding-type DED process. Wang et al. [14] optimized a laser-engineered net shaping (LENS) process by selecting process parameters that maintain a consistent size of the molten pool from their heat transfer simulation. Crespo et al. [15] formed a process map to predict the microstructure of the deposited material according to the scanning speed of laser and interpass time via heat transfer analyses. By controlling the cooling rate, uniform hardness associated with the formation of a microstructure can be achieved [23].

Interpass time is one of the important process strategies in the DED process to passively control the interpass temperature. Interpass time is defined as the time period between the end of the deposition of a bead and the start of the next bead [24]. Interpass temperature refers to the temperature of the deposition area between deposited passes [25]. Denlinger et al. [20] found that the interpass time between planar layers has influenced the formation of the residual stress of substrate during a laser-powder DED process. Babu et al. [1] re-simulated the DED process conducted by Denlinger et al. [20] with the inclusion of a metallurgical model and obtained a similar trend of residual stresses according to the change of interpass time. It is critical to maintain a suitable interpass temperature because it influences the formation of the microstructure, porosity, geometric accuracy, and residual stresses within deposited parts [26,27,28,29].

The build rate is relatively higher for a wire feeding-type DED process than a powder feeding-type DED process. Hence, control of the temperature, particularly for a wire feeding-type DED process, is important to prevent excessive heat accumulation within the specimen [30,31]. Heat accumulation during the continuous deposition of material results in inconsistency in the dimension of deposited material and possible bead collapse [32,33]. Lee et al. [34] experimentally showed that increasing the interpass time has mitigated the part slumping problem during the deposition of a wall structure using a wire feeding-type DED process. A constant interpass time was introduced to passively control the temperature of the specimen during the deposition process [18]. However, selection of the constant interpass time still requires intensive preliminary tests, and it may not solve the heat accumulation problem [35,36]. Montevecchi et al. [33] proposed an algorithm to computationally extract a suitable interpass time for multi-layer deposition using the wire-arc additive manufacturing process from heat transfer finite element analyses (FEAs). Although it is efficient, the implementation of the algorithm using Matlab into different FEA codes is difficult. The effect of proper interpass time for planar layer deposition is yet to be investigated.

In this paper, three-dimensional finite element (FE) models are developed for multi-bead planar layer deposition using a wire feeding-type DED process. A simple and practical methodology based on regression analysis is proposed to investigate the proper interpass time for maintaining interpass temperature during the DED process. From the estimated proper interpass time, a selected proper interpass time scheme (PITS) is suggested for practical implementation. The estimated depths of melt pool and heat-affected zones (HAZs) induced using a constant interpass time scheme (CITS) and selected PITS are compared and discussed. Finally, uncoupled thermo-mechanical FEAs are performed to further evaluate the influence of CITS and selected PITS on the displacement and thermal stress induced within the specimen.

## 2. Analysis of Wire Feeding-Type DED Process

### 2.1. Wire Feeding-Type DED Process

**Figure 1:**
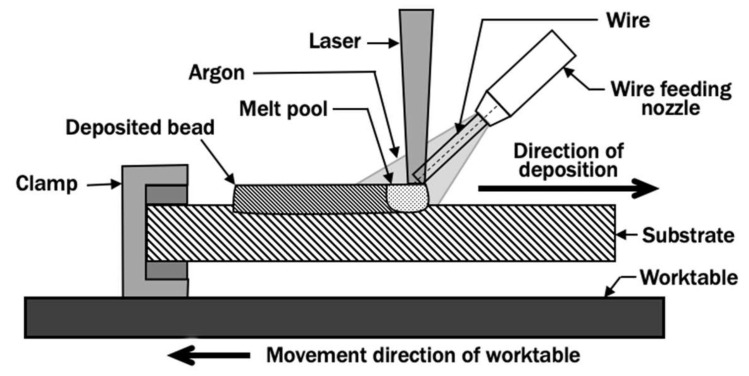
Schematic diagram of a front feeding directed energy deposition (DED) process.

The wire feeding-type DED process applied in this paper is based on the system developed by Kim et al. [37]. The DED system consists of a laser system, a numerically controlled worktable, a wire feeding system, a shielding gas supply system, and a computer control system. The Ytterbium fiber laser is configured to have a top-hat distribution beam with 1.5 mm diameter, measured at the substrate, to serve as a heat source to melt metallic wire feedstock with a wire diameter of 0.9 mm. The power of laser P and travel speed of worktable v are adjusted to 1.5 kW and 8 mm/s, respectively, during the planar layer deposition. The DED system applies a front feeding approach, such that the wire is fed in front of the melt pool in the direction of deposition, as illustrated in Figure 1. Argon gas is used as the shielding gas to prevent the oxidation of a deposited bead and substrate. Titanium alloy Ti–6Al–4V is selected as the material for both the wire and substrate. The cross-section profile of a bead deposited using these process parameters has been experimentally quantified and reported in [38].

### 2.2. Finite Element Models

**Figure 2:**
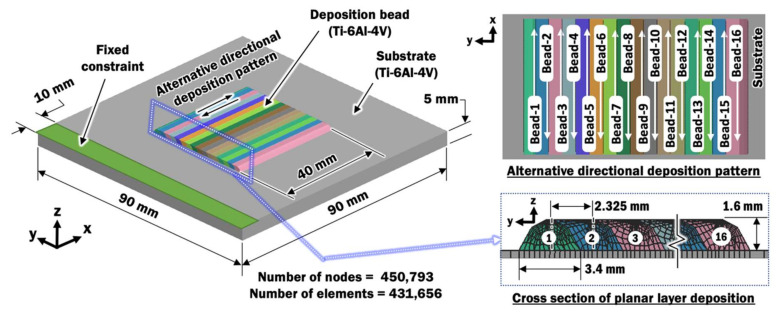
Three-dimensional finite element model [12].

In order to evaluate the distributions of temperatures, vertical displacements, and residual stresses, as well as the estimated depth of HAZs, a thermo-mechanical finite element (FE) model is required. Figure 2 illustrates the three-dimensional finite element (FE) model with sixteen deposited beads lying side-by-side on a 90 mm × 90 mm substrate. These multiple deposited beads that form a flat and wide layer on top of the substrate are known as planar layer deposition. The substrate is clamped on one side of an edge such that the other edges of the substrate are free to displace. The length of each deposited bead is 40 mm.

The planar layer deposition is made using an alternative directional deposition pattern, such that the subsequent bead is deposited from the side where the previous bead deposition ends. The time between the end of a deposition and the start of the subsequent deposition is known as the interpass time [24]. Interpass time is provided for the wire feeding nozzle and laser to orientate to a new position while allowing deposited beads to cool passively.

#### 2.2.1. Overlapping Bead Profile for Planar Layer Deposition

**Figure 3:**
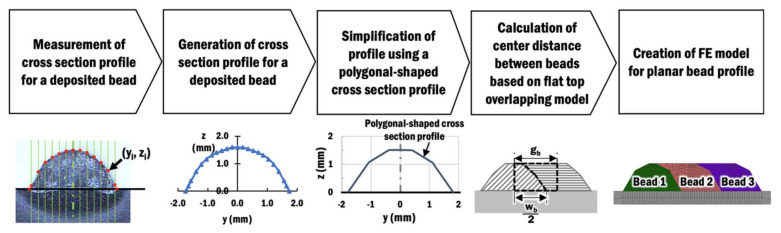
Procedure to model a polygonal-shaped cross-section profile of a multi-bead planar layer [12].

The main intent of constructing a planar layer deposition in a DED process is to obtain a layer with uniform thickness to build upon. Therefore, a flat layer is desired as a result of the deposition process. However, the shape of the bead for a wire-feeding type DED process should be properly modeled due to its size being significantly larger as compared to those of a powder-feeding type DED process. Several overlapping models have been proposed by researchers [39,40,41,42,43] to estimate an optimum center distance between adjacent beads that would produce a uniform thickness profile for the planar layer deposition of a wire feeding-type DED process. The cross-section profile of a single bead with respect to the specific deposition process parameters is required for these overlapping models to be applicable. The basic assumption made in these models is that the overlapped volume between beads is equal to the volume of materials that fill in the valley in between beads in order to form a flat and uniform profile [39,40,41]. In this study, an overlapping bead profile is modeled based on the procedure illustrated in Figure 3.

**Figure 4:**
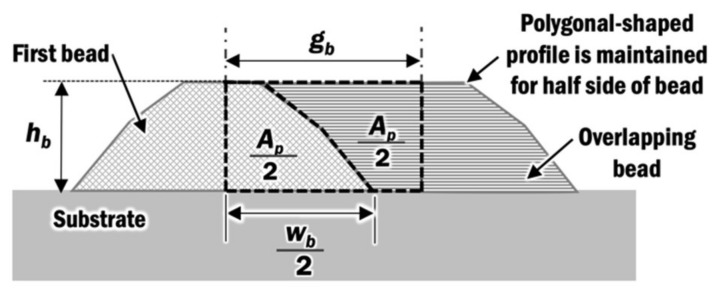
Flat top planar layer profile of multi-bead planar layer.

First, the cross-section profile of a single deposited bead is obtained from the experiment. The cross-section profile for a laser power of 1.5 kW and travel speed of 8 mm/s has been obtained and generated, as shown in [38]. The measured cross-section profile of a single bead is constructed using a simplified polygonal-shaped cross-section profile in a finite element model [12]. The polygonal-shaped cross-section profile is applied because it inherits the simplicity of a rectangular bead profile while preserving the characteristics of a curved-shaped bead by having an approximated wetting angle and height of a bead. A good mesh can be created easily and extendable for both planar layer deposition and multi-layer deposition. The center distance between beads *g_b_* is predicted by Equation (1) to form a flat top planar layer profile in Figure 4, which is based on the basic assumption in those overlapping models.

(1) $$g_{b}=\frac{A_{P}}{h_{b}}$$

where *A_p_* is the cross-sectional area of a polygonal-shaped bead in unit mm^2^, and *h_b_* is the height of the bead in unit mm.

The center distance-to-width ratio *g_w_* is determined by Equation (2):

(2) $$g_{w}\;=\;\frac{g_{b}}{w_{b}}$$

where *w_b_* is the width of the bead in unit mm.

In this study, the optimum distance between beads and distance-to-width ratio are estimated as in Table 1, for the combination of process parameters at a laser power of 1.5 kW and travel speed of 8 mm/s. The determined distance-to-width ratio is within the range of 0.637 to 0.738 derived by several researchers [39,40,41,42,43].

#### 2.2.2. Heat Source Model

**Figure 5:**
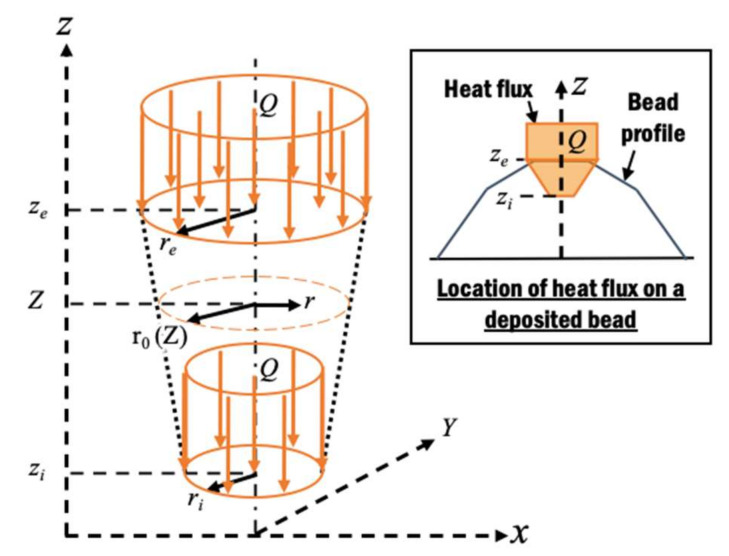
Top-hat distribution of heat flux with conical penetration model on a deposited bead [44,45].

The laser beam heat is represented by a volumetric top-hat distribution of heat flux *Q* with conical penetration depth *d_p_* [12] shown in Figure 5, as given by the mathematical models in Equations (3) and (4):

(3) $$Q\left(r,Z\right)=\left\{\begin{array}{cc} \frac{3\eta P}{\pi d_{p}\left(r_{i}{}^{2}+r_{e}r_{i}+r_{e}{}^{2}\right)} & ,\;\;\;\;\;\;\;\;\;\;\;r\leq r_{0}\;\mathrm{and}\;z_{i}\leq Z\leq z_{e}\\ 0 & \;,\;\;\;r>r_{0}\;\mathrm{or}\;z_{i}>Z\;\mathrm{or}\;Z>z_{e} \end{array}\right.$$

(4) $$r_{o}\left(Z\right)=r_{e}+\left(\frac{r_{i}-r_{e}}{z_{i}-z_{e}}\right)\left(Z-z_{e}\right)$$

where *r* is the radial distance from the center of the beam, *Z* is the z-coordinate relative to the frame of the beam, *η* is the efficiency, *r_e_* is the effective beam radius at the surface of the bead, *r_i_* is the effective beam radius at distance *d_p_* from the surface of the bead, *z_e_* is the z-coordinate at the surface of the bead, *z_i_* is the z-coordinate at distance *d_p_* from the surface of the bead, and *r_o_* is the effective radius at arbitrary z-coordinate.

For the DED system used in this paper, the heat flux model has been calibrated according to the dimension of the heat-affected zone (HAZ) in [38]. The calibrated parameters applied in this paper are summarized in Table 2. The heat flux model is implemented as a moving heat source in commercial FE code Sysweld v2016.0.

#### 2.2.3. Material Properties and Boundary Conditions

**Figure 6:**
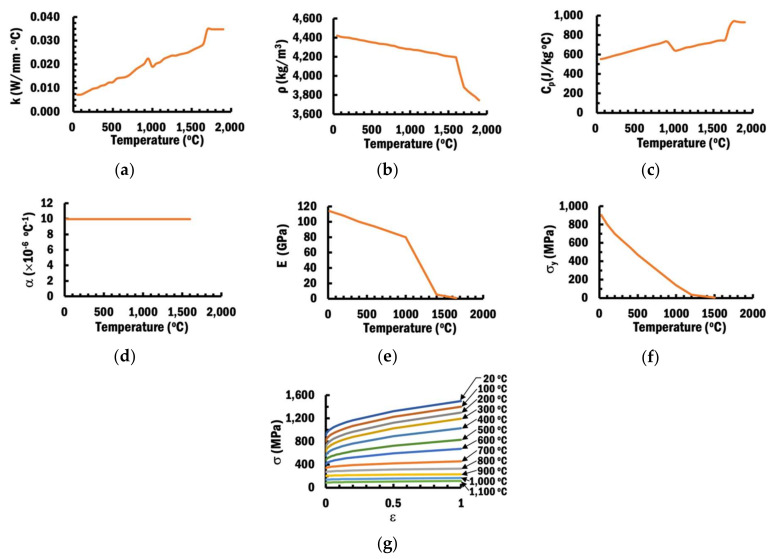
Temperature-dependent material properties of Ti–6Al–4V: (**a**) Thermal conductivity [46]; (**b**) Density [46]; (**c**) Specific heat [46]; (**d**) Coefficient of thermal expansion [47]; (**e**) Modulus of elasticity [47]; (**f**) Yield strength [48]; (**g**) Stress–strain curve [47].

In order to simulate a DED process that involves high temperature, the selected material Ti–6Al–4V for both substrate and beads are assumed to be temperature-dependent material properties in the numerical analysis for better accuracy [12]. Figure 6 shows the temperature-dependent material properties of Ti–6Al–4V, which were obtained from various references [46,47,48]. Poisson’s ratio of Ti–6Al–4V is fixed at 0.342 [49].

The ambient temperature is assigned as 20 ℃. The initial temperature for substrate and beads is set equal to the ambient temperature. An inert gas such as argon is applied directly to the part during the DED process to shield the process zone from oxidation. Based on the empirical correlation model for an impinging jet from a single round nozzle [50], the coefficient of convection for the upper surface of substrate and deposited beads are predicted as a constant value of 46.3 W/m^2^·K during the deposition process. Natural convection is assumed for the side and lower surfaces of the substrate for entire deposition and cooling process. Coefficients of natural convection as a function of temperature at different substrate surfaces are calculated by estimating the average Nusselt numbers *Nu_L_* from recommended correlations in Equations (5)–(7) [50]:

(5) $$Nu_{L}=0.52Ra_{L}^{1/5}\mathrm{for}\;\mathrm{lower}\;\mathrm{surface}$$

(6) $$Nu_{L}=0.68+\left[\frac{0.670Ra_{L}^{1/4}}{{\left(1+{\left(0.492/Pr\right)}^{9/16}\right)}^{4/9}}\right]\mathrm{for}\;\mathrm{side}\;\mathrm{surface}\;$$

(7) $$Nu_{L}=0.54Ra_{L}^{1/4}\mathrm{for}\;\mathrm{upper}\;\mathrm{surface}\;$$

where *Ra_L_* and *Pr* are the Rayleigh number and the Prandtl number, respectively. A temperature-dependent emissivity of solid Ti–6Al–4V and Stefan–Boltzmann constant of 5.67 × 10^−8^ W/(m^2^·K^4^) is adopted to model the thermal heat loss via radiation [46].

## 3. Proposed Estimation of Proper Interpass Time Using Regression Analysis

**Figure 7:**
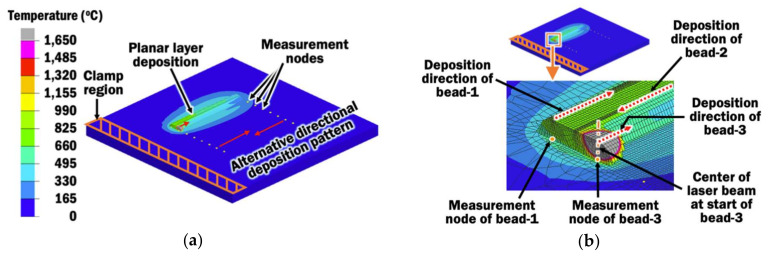
(**a**) Heat transfer FEAs during simulation using different interpass time of 1 s; (**b**) Location of measurement nodes on the substrate for determining the interpass temperature.

A simple and practical methodology based on regression analysis is proposed to investigate the proper interpass time from three results of heat transfer FEAs for maintaining desirable interpass temperature during the planar layer deposition of the DED process. First, three FEAs using interpass times of 1 s, 2 s, and 3 s, respectively, are performed on the FE model described earlier, as shown in Figure 7. Short interpass times are used for planar layer deposition because the cooling of beads is rapid when beads are directly deposited on the large surface of the substrate.

From the temperature histories of FEAs, interpass temperatures can be evaluated. Interpass temperature is defined as the temperature of the deposition area between deposited passes [25]. In order to obtain a more precise control of interpass temperature in this study, interpass temperature *T_ip_* is recorded at a measurement node for each bead on the substrate, after the end of each interpass time. The location of the measurement node for a bead lies at the top surface of the substrate and coincides with the center of the laser beam at the start of deposition of the respective bead, as shown in Figure 7.

**Figure 8:**
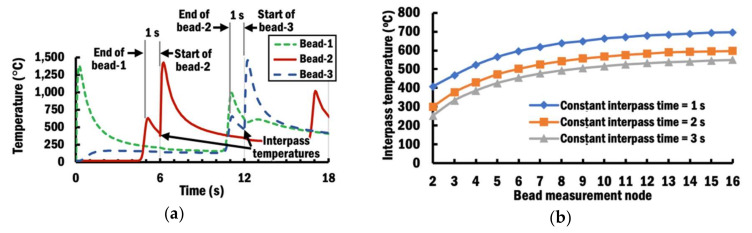
(**a**) Interpass temperatures recorded from temperature histories of 1 s interpass time at the measurement nodes for bead-1, bead-2, and bead-3; (**b**) Interpass temperature using different constant interpass times during planar layer deposition.

Figure 8a illustrates the interpass temperatures recorded at the measurement nodes for bead-1, bead-2, and bead-3 from temperature histories of FEA with an interpass time of 1 s.

From the temperature histories, temperature rises and falls before it rises again to the highest temperature for each bead measurement node except for bead-1. The first rise in temperature is due to heat being transferred during the deposition of the preceding bead to the nearby measurement node. The substrate cools down during the period of the interpass time, which results in falling temperature. The interpass temperature for each bead measurement node is taken at the lowest temperature during the first fall in temperature. After the period of the interpass time ends, the temperature rises to the highest because the deposition of a new bead occurs directly on top of the respective bead measurement node.

**Figure 9:**
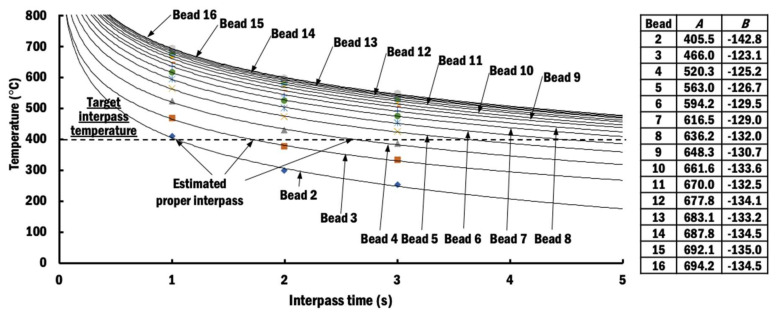
Estimated temperatures of measurement nodes and corresponding constants of regression equation for different beads with respect to interpass time.

Figure 8b shows the change of interpass temperatures at different beads of planar layer deposition using different interpass times. From the results in Figure 8b and based on the temperature decaying characteristics of cooling, the interpass temperatures *T_ip_* for each bead are correlated with interpass times *t_ip_* by logarithmic regression, as shown in Equation (8):

(8) $$T_{ip}\;=\;A+B\;\log_{e}\;\left(t_{ip}\right)$$

where *A* and *B* are constants of the regression equation. Figure 9 illustrates the estimated temperature curves using the proposed logarithmic regression.

By solving Equation (8), the proper interpass time that maintains a target interpass temperature *T_t_* is evaluated in Equation (9). The target interpass temperature for Ti–6Al–4V is assumed to be 400 °C in this study, as recommended [26,27,28,29].

(9) $$t_{ip}\;=\;e^{\left(\frac{T_{t}-A}{B}\right)}$$

**Figure 10:**
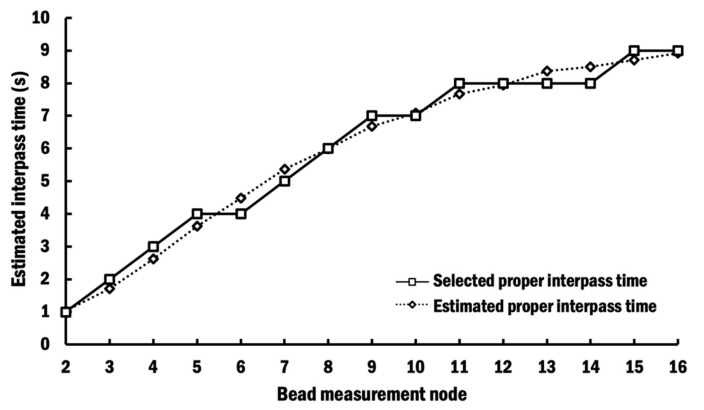
Estimated and selected proper interpass time for different beads.

Figure 10 shows proper interpass times estimated for different beads in order to maintain the target interpass temperature. Selected proper interpass times after the deposition of each layer are rounded to the nearest second with minimum value of 1 s, as indicated by solid black line in Figure 10, for practical implementation during a DED process. They are collectively referred to as the selected proper interpass time scheme (selected PITS).

Using the proposed methodology, no integration of the algorithm between two types of commercial software is required. Furthermore, it can be easily visualized and adjusted by the researcher. The estimated proper interpass time curve in Figure 10 shows a sigmoidal trend, similar to those estimated by Montevecchi et al. [33]. The same trend indicates that the proposed methodology to estimate proper interpass time is proper.

## 4. Results and Discussion

### 4.1. Temperature Distributions

**Figure 11:**
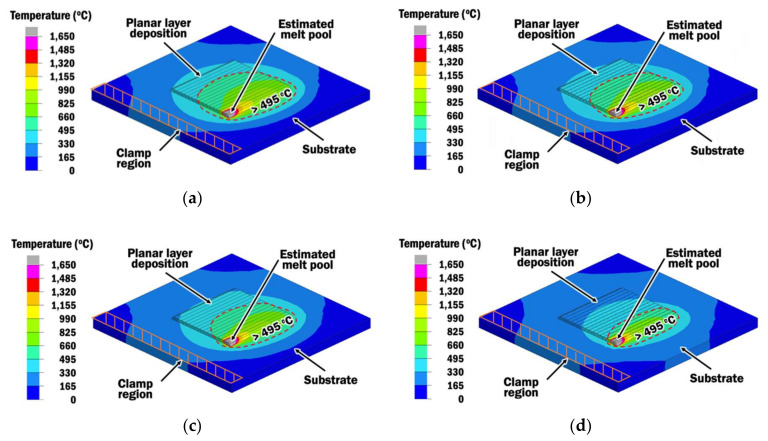
Temperature distributions during the end of deposition of bead-16 from heat transfer FEAs for different interpass times: (**a**) constant interpass time scheme (CITS) of 1 s; (**b**) CITS of 2 s; (**c**) CITS of 3 s; (**d**) selected proper interpass time scheme (PITS).

The influence of interpass time on temperature distributions of planar layer depositions is investigated using results of transient heat transfer FEAs. Figure 11 shows the temperature distributions of planar layer depositions using different constant interpass time schemes (CITS) of 1, 2, and 3 s and a selected proper interpass time scheme (PITS) at the end of deposition of bead-16.

For selected PITS, the interpass time shown in Figure 10 is applied before the deposition of the respective bead, as being represented by the bead measurement node. Regions on the top surface of the specimens with high temperature (higher than 495 °C) are circled for quick comparisons. The implementation of a longer interpass time results in a size reduction of the circled region of high temperature. It reveals that there is a noticeable variation in temperature distribution, although a small change of 1 s is applied as the interpass time. Hence, a wire feeding-type DED process using different interpass times has influenced the interpass temperatures. Evolutions of the interpass temperatures, the depth of the melt pool, and the depth of the heat-affected zone with incremental bead depositions are discussed in the following sections.

#### 4.1.1. Interpass Temperature Using Selected Proper Interpass Time Scheme

**Figure 12:**
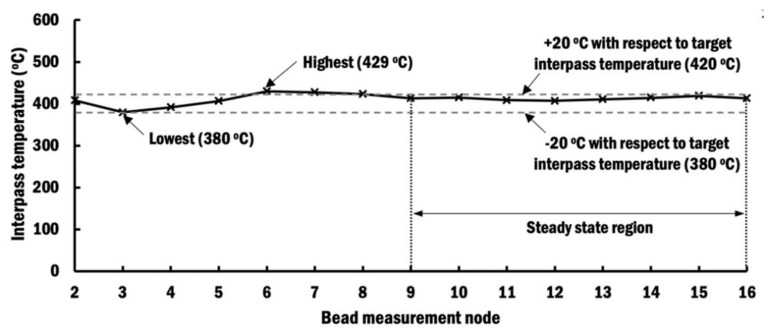
Interpass temperatures of planar layer depositions using a selected proper interpass time scheme.

A heat transfer FEA is performed using the selected proper interpass time scheme. From the result of heat transfer FEA, estimated interpass temperatures at the measurement node of each bead are recorded, as shown in Figure 12. The highest and lowest interpass temperatures recorded are 429 and 380 °C, respectively. This represents a variation of interpass temperature in the range between −20 and +29 °C from the target interpass temperature of 400 °C. The variation can be attributed to the selection of interpass time that has been rounded to an integer. From Figure 12, it shows the interpass temperatures at the ninth bead measurement node and onwards are successfully kept in a steady state ranging within ±20 °C from the target interpass temperature. The steady-state region of the interpass temperature is achieved when the number of beads deposited on the substrate increases. This is because the variation of temperature within the substrate has become stable.

In comparison to those curves in Figure 8b that apply constant interpass time schemes (CITS), it is clearly shown that the selected PITS has managed to control the interpass temperature to be near the desired temperature of 400 °C. The selected PITS has applied the minimum 1 s interpass time at the beginning to rapidly increase the interpass temperature of the substrate. Interpass times of selected PITS are progressively increased to maintain the interpass temperatures near the target temperature. This is because the temperature of the substrate has risen after several depositions of beads, and it requires a longer interpass time to passively cool it down to the target temperature. Therefore, these FEA results indicate that the proposed estimation of a proper interpass time using regression analysis is capable of controlling the interpass temperature within a desired range during the planar layer deposition of a wire feeding-type DED process.

#### 4.1.2. Depth of Melt Pool and Heat-Affected Zone

**Figure 13:**
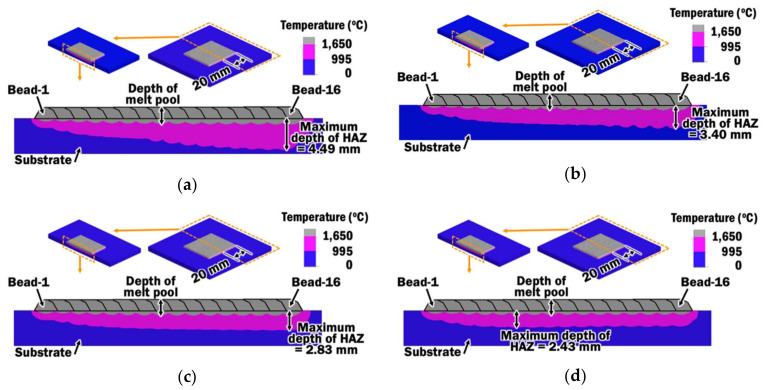
Distributions of peak temperature for planar layer deposition using different interpass time schemes: (**a**) CITS of 1 s; (**b**) CITS of 2 s; (**c**) CITS of 3 s; (**d**) Selected PITS.

From the results of heat transfer FEAs, distributions of peak temperature throughout the DED planar layer deposition at the sectional plane across the middle of beads and substrate are obtained, as illustrated in Figure 13. The distributions of peak temperature are used to estimate the formation of a melt pool and heat-affected zone (HAZ) during the wire feeding-type DED process. The melt pool is estimated as the gray-colored region with temperature exceeding 1650 °C, which is the liquidus melting temperature of Ti–6Al–4V [51]. The HAZ of Ti–6Al–4V is associated with the change in microstructure from an alpha-beta phase to a body-centered cubic structured beta phase due to peak temperature. Hence, the estimated HAZ is referring to the pink-colored region that has a peak temperature between the beta transus temperature of 995 °C for Ti–6Al–4V [52] and liquidus temperature of 1650 °C.

**Figure 14:**
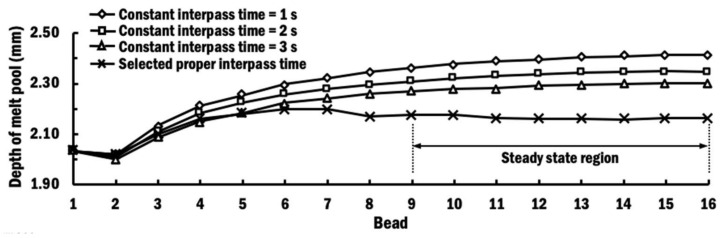
Comparison of depth of melt pool during planar layer deposition process of different beads using different schemes of interpass times.

The depth of the melt pool with respect to each deposition of bead, as plotted in Figure 14, is measured as the maximum vertical distance from the top surface of the respective bead to the boundary between the melt pool and the HAZ. It shows that the depth of the melt pool grows when the number of bead depositions increase for planar layer deposition using CITS. This is due to the fact that the thermal conductivity of Ti–6Al–4V increases upon raising the temperature of substrate. Therefore, heat from the laser beam spreads farther to a deeper melt pool when the temperature of the substrate is higher. This phenomenon is more obvious in the case of a low interpass time of 1 s. The trends for the depth of the melt pool using different CITS are similar to those of interpass temperatures using different CITS, as shown in Figure 8b.

In the case of planar layer deposition using selected PITS, the depth of the melt pool grows from bead-1 and reaches the maximum at bead-6. The depth of the melt pool is in the steady-state region from the deposition of bead-9 and onwards, when the interpass temperature is close to the target interpass temperature. The variation of depths of melt pools at the steady-state region is small and negligible, which is in the range of below 17 µm. This observation is consistent with the steady-state region of interpass temperatures. The estimated depth of the melt pool for all beads deposited using selected PITS is greater than the height of beads of 1.6 mm. This observation ensures that these beads and substrate are properly fused during planar layer deposition using the selected PITS. Thus, the control of interpass temperatures using the selected PITS is useful to control the formation of the melt pool while ensuring sufficient fusion between the beads and substrate.

The maximum depth of HAZ is defined as the maximum vertical distance from the substrate to the lowest point of the HAZ region. During planar layer deposition using CITS, it is observed that the estimated HAZ region continuously deepens with increasing bead depositions. All the maximum depths of the HAZ for planar layer depositions using different CITS are identified near bead-16. In contrast, the maximum depth of the HAZ for planar layer deposition using the selected PITS is found near bead-6 and converged to a steady-state depth of HAZ for the further deposition of beads. This observation coincides with the highest estimated interpass temperatures at bead-6, as shown in Figure 12. Hence, the interpass temperature at each measurement node has directly influenced the formation of the melt pool and HAZ. The application of PITS to control the interpass temperatures shows promising contribution to control the growing HAZ and microstructure transformation to a manageable depth when a wide planar layer deposition is required.

### 4.2. Thermo-Mechanical Characteristics during Planar Layer Deposition

In order to investigate the evolution of thermo-mechanical characteristics induced within deposited beads of planar layer deposition using different interpass time schemes, vertical displacement and residual stresses are evaluated from results of thermo-mechanical FEAs. Thermo-mechanical characteristics between a constant interpass time scheme (CITS) of 1 s and selected proper interpass time scheme (PITS) are compared to assess the suitability of the proposed selected PITS for the fabrication of a Ti–6Al–4V planar layer.

#### 4.2.1. Vertical Displacement

**Figure 15:**
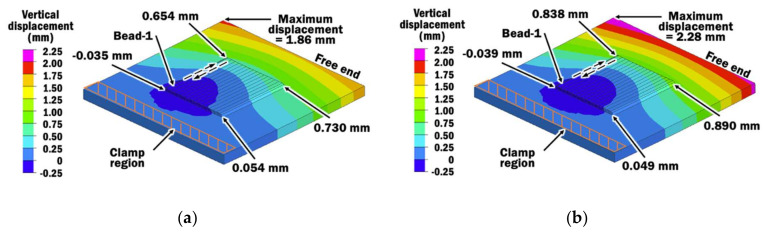
(**a**) Vertical displacement distribution at the end of deposition of bead-16 using CITS of 1 s; (**b**) Vertical displacement distribution at the end of deposition of bead-16 using selected PITS.

**Figure 16:**
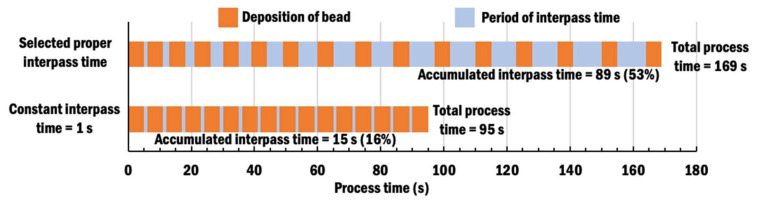
Process time according to different interpass time schemes.

Figure 15a,b shows vertical displacement distributions estimated at the end of deposition of bead-16 using CITS of 1 s and selected PITS, respectively. By comparing vertical displacements at four corners of the planar layer, a similar trend of vertical displacements is observed for depositions using different interpass time schemes. A small portion of the specimen near the start point of bead-1 deposition and clamp region undergoes downwards displacement. The maximum vertical displacement is located at the corner near to bead-1 at the free end. However, the selected PITS produces a specimen with a greater amount of vertical displacement than the CITS of 1 s. This is due to the fact that deposition using the selected PITS requires a longer interpass time for cooling. Thus, the specimen with deposition using the selected PITS has more time to displace before a new bead is deposited, as illustrated in Figure 16.

**Figure 17:**
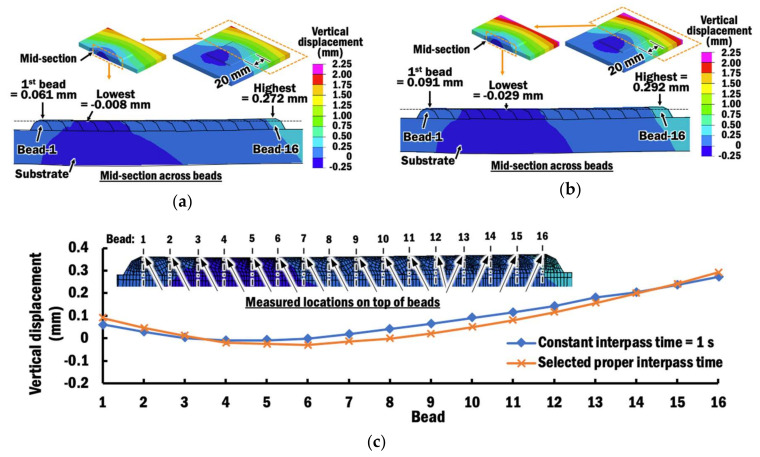
(**a**) Vertical displacement distribution at measured mid-sections during the end of deposition of bead-16 using CITS of 1 s; (**b**) Vertical displacement distribution at measured mid-sections during the end of deposition of bead-16 using the selected PITS; (**c**) Comparison of vertical displacement profiles of planar layer at the measured mid-sections for different interpass time schemes.

In order to further investigate the influence of interpass time schemes on the surface profile across beads of the planar layer at the end of deposition of bead-16, vertical displacements on the top of each bead are measured at the mid-section of the planar layer, as shown in Figure 17. It is observed that the planar layer tends to curve upwards at the sides to form a U-shape, regardless of the interpass time schemes. The differences in vertical displacement on the mid-section of a planar layer deposited using selected PITS and CITS are ranging between 4.2 and 44.6 µm. The largest difference occurs at bead-9. However, these variations are small compared to the height of the deposited planar layer and will have a negligible effect when the second planar layer is deposited on top of it. Hence, the proposed selected PITS is acceptable for planar layer deposition.

#### 4.2.2. Induced Residual Stress

**Figure 18:**
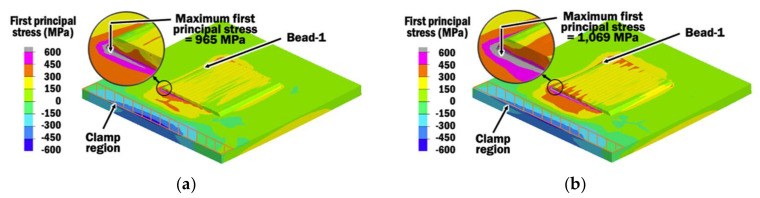
(**a**) Vertical displacement distribution at the end of deposition of bead-16 using CITS of 1 s; (**b**) Vertical displacement distribution at the end of deposition of bead-16 using the selected PITS.

Figure 18a,b shows the residual stress distributions induced within specimens during the end of deposition of bead-16 using a CITS of 1 s and selected PITS, respectively. In order to evaluate the types of stresses and possibility of a crack region due to these induced residual stresses during planar layer depositions using different interpass time schemes, the first principal stress is estimated from the results of thermo-mechanical FEAs. Compression is observed at the clamp region, while the remaining part of the substrate and planar layer are undergoing tension for both cases of interpass time schemes. Compression occurs at the clamp region because the thermal expansion and displacement of the substrate are being suppressed by the clamp when the temperature increases during planar layer deposition.

The maximum stress is estimated at the substrate near the corner of the planar layer of bead-1. It is noted that the stresses near the maximum stresses in both cases of interpass time schemes have changed tremendously from below 450 MPa to over 950 MPa. These sharp changes in residual stresses are due to the fact that there are singularities problems in FEAs caused by the sharp 90° corners between the substrate and the planar layer of the FEA models. Thus, these maximum residual stresses estimated in FEAs should be excluded from discussion. Nevertheless, the location of the maximum first principal stress indicates a region where cracking may start to occur. Overall, the planar layers are undergoing tension less than 450 MPa.

**Figure 19:**
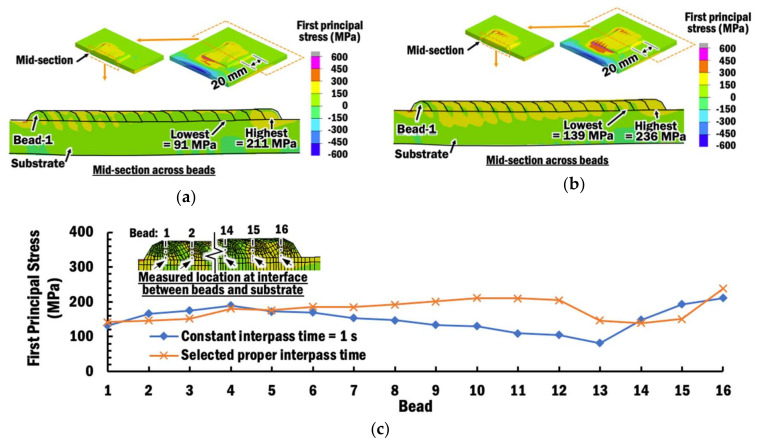
(**a**) First principal stress distribution at the end of deposition of bead-16 using CITS of 1 s; (**b**) First principal stress distribution at the end of deposition of bead-16 using selected PITS; (**c**) Comparison of first principal stress across the multi-beads planar layer measured at the interface between the beads and substrate of mid-sections for different interpass time schemes.

**Figure 20:**
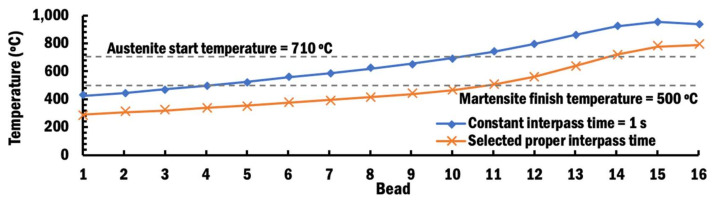
Temperatures estimated for measured locations at the interface between the beads and substrate of the mid-section for different interpass schemes.

In order to compare the influence of interpass time schemes on the formation of residual stresses within beads of the planar layer at the end of deposition of bead-16, the first principal stresses at the intersection points between the center of the beads and the surface of the substrate are measured at the mid-section of the planar layer, as shown in Figure 19. Figure 19 shows that the highest residual stresses of 211 MPa and 236 MPa are induced at bead-16 of the mid-section using a CITS of 1 s and selected PITS, respectively. This observation is due to the thermally expanded bead-16, which is still hot after being deposited, stretching the surface of the substrate. Several beads adjacent to bead-16 experience lower stress because they have started phase transformation when being re-heated to a temperature above 710 °C [47], and this phenomena is known as stress relaxation [20], as shown in Figure 20.

**Figure 21:**
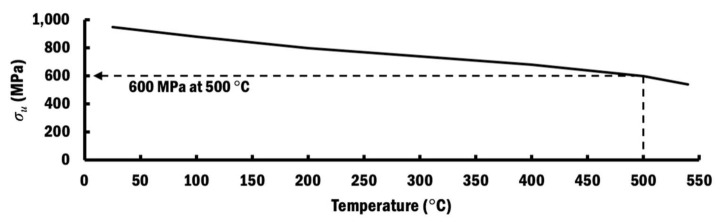
Ultimate strength of Ti–6Al–4V at elevated temperature [48].

In the case of the selected PITS, the residual stress slowly increases from 142 MPa at the measured location of bead-1 to 210 MPa at the measured location of bead-11. This steady increment is because larger thermal expansion occurs at higher temperature and results in tensile residual stress. These residual stresses are not influenced by any further phase change when the temperature of the measured location at the interface between the beads and substrate of the mid-section is lower than the martensite finish temperature of 500 °C [47] during the cooling process. However, only beads 1 to 4 have temperatures below 500 °C in the case of CITS, even though deposition of bead-16 has ended. In the case of CITS, the residual stress increases from 132 MPa at bead-1 to 189 MPa at bead-4. By comparing the residual stresses at measured locations of bead-1 to bead-4 for both cases, it shows that the selected PITS induces a slightly lower tensile stress. This can be attributed to the slightly larger displacement during planar layer deposition using the selected PITS. These residual stresses are much lower than the ultimate strength of 600 MPa at an elevated temperature of 500 °C for Ti–6Al–4V [48], as shown in Figure 21. Therefore, cracks at the interface between the beads and the substrate are unlikely to happen.

Maximum tensile residual stresses are usually found at the interface between the beads and substrate, although the residual stresses vary at different locations of a multilayer three-dimensional printed part [5]. Therefore, an investigation of residual stresses using the deposition of a single layer of 16 beads on the substrate will provide preliminary information for a successful deposition process, especially when the deposition is carried out without preheating. This kind of investigation will be of greater importance when beads are deposited on a dissimilar metallic substrate [53]. With the control of depth of HAZ, the risk of cracks can be further minimized.

From these viewpoints, the selected proper interpass time scheme should be applied because it provides better control of growth in the depth of the melt pool and HAZ without inducing excessive displacement and residual stress within a planar layer deposited using a wire feeding-type DED process.

## 5. Conclusions

In this paper, a simple and practical methodology based on regression analysis was proposed to estimate the proper interpass time from several heat transfer finite element analyses to maintain the interpass temperature during a DED planar layer deposition. Control of the interpass temperature is crucial to prevent problems due to excessive heat accumulation. A three-dimensional finite element (FE) model was developed for multi-bead planar layer deposition by a wire feeding-type DED process. A polygonal-shaped cross-section profile of a single bead and overlapping model were implemented for the construction of the multi-bead planar layer in the FE model. From the estimated proper interpass time, criteria for practical implementation were proposed to select a proper interpass time scheme.

As the result of implementing the proposed methodology, the interpass time was progressively increased with the number of beads. The interpass temperatures were successfully maintained within the range of −20 and 29 °C from the target interpass temperature of 400 °C. The proposed PITS reduces the overall process time when compared to a CITS of 9 s, which will converge slowly with large temperature variation to the same target interpass temperature. Thermo-mechanical analyses revealed that the depths of HAZ had peaked at 2.43 mm after the deposition of bead-6. The depths of the HAZs were kept within a steady-state region with no cracks being predicted at the interface between the beads and substrate. Hence, the proposed PITS based on regression analysis is effective for the mitigation of growing HAZ without severely inducing large displacement and residual stresses when a wide planar layer deposition is required.

This methodology can be extended for a multi-layer deposition of the DED process. In order to create a multilayer three-dimensional part, the proposed selected PITS can be applied for the subsequent layer of deposition. The period of interpass time required to maintain a constant temperature is expected to increase when the number of layers is increased due to the heat accumulation in the preceding layer and reduced heat loss via conduction to substrate. However, the proposed PITS may not be suitable for a large or complex shape object, in which the thermal history of previous deposition has a minimal influence on the next deposition of a bead or a layer.

## Figures and Tables

**Table 1:** Center distance between beads and distance-to-width ratio for *P* = 1.5 kW and *v* = 8 mm/s.

Power of Laser (*P*, kW)	Travel Speed (*v*, mm/s)	Cross-Section Area of Polygonal-Shaped Profile (*A_p_*, mm^2^)	Height of Bead (*h_b_*, mm)	Center Distance between Beads (*g_b_*, mm)	Center Distance-to-Width Ratio (*g_w_*)
1.5	8	3.720	1.6	2.325	0.684

**Table 2:** Calibrated parameters for heat flux model [38].

Power of Laser (*P*, kW)	Efficiency of Laser (*η*, %)	Penetration Depth (*d_p_*, mm)	Effective Beam Radius at Surface of Bead (*r_e_*, mm)	Effective Beam Radius at *d_p_* (*r_i_*, mm)
1.5	60	0.2	0.750	0.375

## Abbreviations

The following abbreviations are used in this manuscript:

DED	Directed Energy Deposition
LENS	Laser Engineered Net Shaping
FE	Finite Element
FEA	Finite Element Analysis
HAZ	Heat Affected Zone
CITS	Constant Interpass Time Scheme
PITS	Proper Interpass Time Scheme
